# A Review of Intraocular Lens Power Calculation Formulas Based on Artificial Intelligence

**DOI:** 10.3390/jcm13020498

**Published:** 2024-01-16

**Authors:** Wiktor Stopyra, David L. Cooke, Andrzej Grzybowski

**Affiliations:** 1 MW-Med Eye Centre, 31-416 Krakow, Poland; wiktorstopyra@gmail.com; 2 Department of Medicine, University of Applied Sciences, 34-400 Nowy Targ, Poland; 3 Great Lakes Eye Care, Saint Joseph, MI 49085, USA; davidlcooke@gmail.com; 4 Department of Neurology and Ophthalmology, College of Osteopathic Medicine, Michigan State University, East Lansing, MI 48824, USA; 5 Institute for Research in Ophthalmology, Foundation for Ophthalmology Development, 61-553 Poznan, Poland

**Keywords:** artificial intelligence, phacoemulsification, intraocular lens power calculation formulas, the Kane formula

## Abstract

Purpose: The proper selection of an intraocular lens power calculation formula is an essential aspect of cataract surgery. This study evaluated the accuracy of artificial intelligence-based formulas. Design: Systematic review. Methods: This review comprises articles evaluating the exactness of artificial intelligence-based formulas published from 2017 to July 2023. The papers were identified by a literature search of various databases (Pubmed/MEDLINE, Google Scholar, Crossref, Cochrane Library, Web of Science, and SciELO) using the terms “IOL formulas”, “FullMonte”, “Ladas”, “Hill-RBF”, “PEARL-DGS”, “Kane”, “Karmona”, “Hoffer QST”, and “Nallasamy”. In total, 25 peer-reviewed articles in English with the maximum sample and the largest number of compared formulas were examined. Results: The scores of the mean absolute error and percentage of patients within ±0.5 D and ±1.0 D were used to estimate the exactness of the formulas. In most studies the Kane formula obtained the smallest mean absolute error and the highest percentage of patients within ±0.5 D and ±1.0 D. Second place was typically achieved by the PEARL DGS formula. The limitations of the studies were also discussed. Conclusions: Kane seems to be the most accurate artificial intelligence-based formula. PEARL DGS also gives very good results. Hoffer QST, Karmona, and Nallasamy are the newest, and need further evaluation.

## 1. Introduction

Phacoemulsification is the most commonly performed ophthalmological surgical procedure [1]. Cataract surgery is often a refractive procedure and not only an extraction technique [2,3]. Patients’ expectations for perfect vision after phacoemulsification are still increasing [4].

However, recent studies have found that only 70–80% of eyes obtained a postoperative refraction within ±0.50 D of the forecasted value [5]. The European Registry of Quality Outcomes reported that the percentage of the prediction error within ±0.5 D after cataract surgery was 73.7% [6]. This is why so many new methods have been developed in recent years [7,8,9,10,11,12]. The accuracy of intraocular lens (IOL) power estimation depends on the accuracy of preoperative biometric data, e.g., the axial length (AL), keratometry (K), and anterior chamber depth (ACD), and on the performance of the intraocular lens (IOL) power calculation formula used [13].

The most widely used IOL power formulas (SRK/T, Hoffer Q, Holladay 1, Haigis) are vergence-based and traditionally classified by generations [14], however, the most popular rating is based on a logical approach [15]. These formulas estimate the effective lens position (ELP) using different parameters, e.g., AL, K, ACD, white to white (WTW) distance, and lens thickness (LT). An accurate ELP forecast, directly dependent on the postoperative ACD evaluation, has been one of the most important limitations to obtain correct refractive results—responsible for about 40% of the prediction error [16]. It has become obvious that there is a need for improved IOL power formula predictions.

Artificial intelligence (AI), as a term, was coined by McCarthy et al. in 1955, however, it is Alan Turing who is assumed to be a father of theoretical computer science and artificial intelligence, since in 1948 he presented many of the main concepts of AI [17]. AI is used in many areas of medicine. In ophthalmology, AI and deep learning, as an AI application, have been applied to visual fields, fundus photography, and optical coherence tomography, obtaining excellent classification performances in the detection of retinopathy (diabetic and prematurity) [18]. They have also evaluated macular edema, the glaucoma-like disc, and age-related macular degeneration well [17,19]. AI refers to techniques for machines that mimic human behavior; machine learning is a subset of AI whereby machines can improve without explicit programming, and deep learning is a subset of machine learning whereby machines can self-train to perform tasks using extensive data sets fed into multilayered neural networks [20].

The FullMonte IOL method was promulgated by Clarke—a pioneer in new methods for IOL power selection. It is no longer operational but it highlighted the value of AI in IOL power calculations. It used a Monte Carlo Markov Chain simulator to produce its refractive predictions [7]. Recently, some new-generation formulas have appeared. They show promising results using the latest methodologies, including AI and a large number of preoperative eye parameters, to calculate the postoperative refractive error [21]. The Kane formula was created using several large data sets from selected high-volume surgeons. It uses a combination of theoretical optics, thin lens formulas, and ‘big data’ techniques to make its predictions [22]. Prediction Enhanced by ARtificial Intelligence and output Linearization created by Debellemanière, Gatinel and Saad (PEARL-DGS) is a thick lens formula based on the prediction of the theoretical internal lens position (TILP), back-calculated from postoperative data [23]. The Ladas Super Formula AI predicts the refractive outcome by using one of five third- or fourth-generation formulas depending on what the literature has shown to be the most accurate formula for that particular combination of AL and K [8]. The Hill-radial basis function (RBF) uses deep learning from a large data set to predict refractive outcomes (a pure AI regression method). This formula adds a validating boundary model by which it refuses to provide a refractive prediction as it is more likely to be inaccurate [21]. The Karmona formula is the result of pure artificial intelligence without Gaussian theoretical components. It is based on two combined models of machine learning called a regularized Bayesian neural network and cubist decision tree [24]. Hoffer Q/Savini/Taroni (Hoffer QST) is an evolution of the Hoffer Q formula to improve its accuracy [24,25]. The Nallasamy formula is based on an ensemble machine-learning model which has recently been introduced [26].

The variables utilized in these formulas are presented in Table 1.

**Figure 1:**
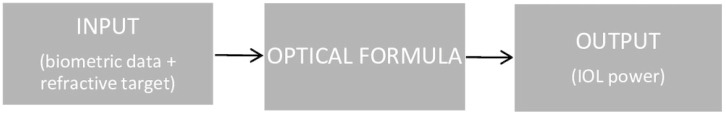
The scheme of IOL power calculation using an optical formula.

**Figure 2:**
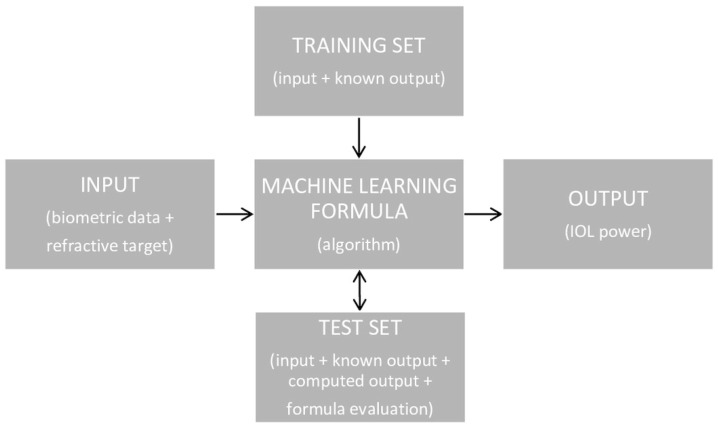
The scheme of IOL power calculation using an AI formula.

The calculation of the IOL’s power using an optical formula is based on a simple scheme, shown in Figure 1. However, this scheme is much more elaborate when using the AI formula (Figure 2). There are two possible AI-based formula structures. The first is most often used, in which AI methods can be designed to either foretell the postoperative spherical equivalent (SE) for a known IOL power and set of biometric variables, while the second can predict the IOL power engrafted given the postoperative SE and biometric data [17].

To the authors’ knowledge no peer-reviewed publications have compared the accuracy of all eight IOL power calculation formulas based on AI. There are studies evaluating two [9,21,27,28,29,30], three [11,21,24,25,31,32], and a maximum of four of them [2,12,26]. The aim of this paper is to compare the correctness of all existing AI IOL power calculation formulas, including hybrids, as an overview of the latest articles.

## 2. Methods

Our methodology follows the Preferred Reporting Items for Systematic Reviews and Meta-Analysis (PRISMA) guidelines. This review considers articles evaluating the accuracy of IOL power calculation formulas based on AI published from 2017 to July 2023. The papers were identified by a literature search of medical and other databases (Pubmed/MEDLINE, Web of Science, Google Scholar, Crossref, Cochrane Library, and SciELO) utilizing the terms “IOL formulas”, “FullMonte”, “Ladas”, “Hill-RBF”, “PEARL-DGS”, “Kane”, “Karmona”, “Hoffer QST”, and “Nallasamy”. First, duplicates were deleted at the screening stage. Then, using abstracts, a preliminary search was performed. Only peer-reviewed papers in English were examined. Studies presented as editorials, protocols, and commentaries were excluded. Finally, the studies with the maximum sample size (>75 patients), the largest number of compared AI IOL power calculation formulas (≥2), and that were the most recent (from the last 6 years) were used. Articles with a risk of bias were not removed, however, the limitations of all studies were discussed in detail. Each procedure was performed twice by 1 author (WS) for accuracy.

Many various tools could be employed to estimate the accuracy of a selected formula. We based our observations on the mean absolute error (MAE) and the percentage of eyes within ±0.5 D.

### 2.1. Formula Findings

Three IOL power calculation formulas based purely on AI are considered in the peer-reviewed literature—Hill-RBF [2,6,7,9,12,22,31,32], Karmona [24], and Nallasamy [27]. They are all independent of Gaussian optics but differ in their mathematical approach. Additionally, five hybrid IOL power calculation formulas (AI and theoretical models combined) are published in the peer-review literature. In order of their development these are the FullMonte Method, the Ladas Super Formula AI, the PEARL-DGS formula, the Kane formula, and the Hoffer QST formula.

### 2.2. The Hill-RBF Formula

Hill-RBF was introduced in 2016 by Warren E. Hill, MD, and was based on RBF, which is similar to a neural network. The Hill-RBF formula is a pure data-driven IOL power calculation method and therefore is free of the restriction and benefit of a lens-position assessment. It uses pattern recognition developed by Matlab and a refined form of data interpolation. Starting with a large number of cases where the biometry and the outcomes are known, RBF is capable of finding distinct patterns in the apparently random cloud of data points. The formula has been optimized for use with variables obtained from the LENSTAR LS 900 (Haag-Streit, Köniz, Switzerland) optical biometer in combination with the Alcon SN60WF (Alcon Laboratories, Fortworth, TX, USA) biconvex IOL for powers from +6.00 D to +30.00 D. It has separately been optimized for IOL powers up to +35.00 D based on a similar biconvex IOL design. For IOL powers from +5.00 D to −5.00 D, it performs best with the biometry data obtained from the aforementioned device and the Alcon MA60MA (Alcon Laboratories, Fortworth, TX, USA) extended-range IOL. The Hill-RBF online calculator may also be utilized with data from other devices, which yield clinically equivalent biometry data compared to the LENSTAR LS 900. It can also be exploited with other biconvex IOL models in the power range of +6.00 D to +35.00 D and other meniscus design IOL models in the power range of +5.00 D to −5.00 D. The formula utilizes a large data set—over 12,000 eyes in version 2.0 and more than 30,000 eyes in version 3.0—which has been available since September 2020. However, Hill-RBF will not provide a refractive prediction if the preoperative parameters are out of bounds, as predictions are likely to be inaccurate. In addition, early versions did not allow the input of a target refraction other than plano [7] The Hill-RBF 3.0 formula uses following variables: AL, K, ACD, LT, WTW, central corneal thickness (CCT), and gender [33].

### 2.3. The Karmona Formula

Karmona—the newest of these IOL power calculation formulas—was designed and programmed in Shiny-RStudio Version 1.1.423 (R Foundation, Boston, MA, USA) between 2018 and 2021 by David Carmona González from Spain. It is the result of pure artificial intelligence without Gaussian theoretical components. It is based on two combined models of machine learning called a regularized Bayesian neural network and cubist decision tree. These were selected from eleven nonlinear regression models (k-Nearest Neighbors, Support Vector Machines with RBF, linear discriminant analysis or with a linear kernel, decision trees, neural networks testing several hidden layers, random forest, Least Absolute Shrinkage and Selection Operator regression, a generalized linear model, multivariate adaptive regression spline, and stochastic gradient boosting) as the most efficient. Then, they were tuned, thereby improving their metrics. Finally, an ensembled model was generated via a machine learning method that combined prediction from separate strategies [24]. The data inputs of Karmona include AL, ACD, K, WTW, and the corneal anterior surface central radius. LT, anterior segment depth (the sum of ACD and LT), posterior corneal central radius, ratio of the back to front corneal surface central radius, and gender are optional variables. Karmona is optimized for anterior keratometry with IOL Master^®^ 700 (Carl Zeiss Meditec, Jena, Germany) and for posterior keratometry with Pentacam^®^ (Oculus Optikgeräte GmbH, Wetzlar, Germany), however, it can apparently be utilized with data from any other device. It uses the A constant as the IOL constant [34]. As a new method, Karmona has been introduced with a database of only 386 eyes, with standard axial lengths (22.0–25.0 mm). As with any other formula based on AI, it is constantly learning, so its predictive volume is expected to continuously improve. The formula has potentially been designed to increase the accuracy in extreme long or short and atypical eyes, since it uses unique data inputs, i.e., the ratio of the back to front corneal surface central radius and anterior segment depth as a separate parameter.

### 2.4. The Nallasamy Formula

In 2022 Li, Stein, and Nallasamy published a formula based on a data set of 5016 eyes. The preoperative input data were obtained from Lenstar LS 900 optical biometers (Haag-Streit USA; EyeSuite software V.i9.1.0.0) at the University of Michigan’s Kellogg Eye Center. Nallasamy et al. utilized an ensemble machine learning model which consisted of two layers. In the first stratum, a group of level-1 learners was taught based on preoperative patient data and IOL power as input data and the postoperative refraction as the target value. The second stratum includes the meta-model, which uses the output of the level-1 learners as its input marks. So, the number of input qualities for the level-2 model is comparative to the number of level-1 models. The output from the level-2 meta-model is the final predicted refraction. As input data, the formula uses five compulsory variables such AL; K; ACD; WTW; LT; and one optional variable, i.e., CCT [27].

### 2.5. The FullMonte Method

The history of Fullmonte is interesting and inspiring, as Gerald Clarke was its pioneer, proposing with Burmeister in 1997 the first neural network for biometric computations [35]. They expanded and trained a neural network to forecast IOL powers utilizing a personalized Holladay program and clinical data from 200 subsequent cases of one surgeon’s outcomes with one model of IOL. They used preoperative AL, both K values, ACD, and LT. The neural network was trained to develop a postoperative refractive error, and the Holladay surgeon factor was continuously enriched utilizing the same outcomes. After the network was successfully trained with the clinical data, it was exploited to count IOL power in a double-masked study [7]. The Fullmonte method was a hybrid. Clarke and Kalpener proposed a combined formula between SRK/T and an AI algorithm called Bayesian Additive Regression Trees, optimized with a Markov Chain Monte Carlo simulator to produce its refractive forecast [36]. Unpublished data have shown a decrease in their standard deviation when confronted with the SRK/T formula. Unfortunately, the FullMonte website is no longer functional.

### 2.6. The Ladas Super Formula AI

In 2015, John G. Ladas, Albert Jun, Aazim Siddiqui, and Uday Devgan introduced the idea of an IOL ‘super formula’, which incorporated the qualities of previous formulas. They considered third-generation formulas (Holladay 1, Hoffer Q, Holladay 1 with Koch adjustment, SRK/T) and a fourth generation formula, i.e., Haigis [8]. Although past formulas were thought of as two-dimensional algebraic equations, they developed a novel way of depicting these formulas as mathematical equations with the potential to be graphed on the *x*, *y*, and *z* axes and rendered them in three dimensions. In this way they provided a framework to analyze these formulas in three dimensions and observe areas of differentiation. Using this observation and peer-reviewed literature, the identified best parts of each of the mentioned IOL methods were selected and an IOL ‘super surface’ was constructed [37]. Retrospective studies have shown that while the SRK/T yields better outcomes for longer eyes [38,39], Hoffer Q achieves more accurate results with very short eyes [14]. Some eyes require more nuance and optimization. It is known that shorter eyes are more difficult to count as the smallest of changes in ELP can alter the IOL power calculations drastically. From this super surface, the ‘super formula’ was derived [8,37]. An interesting approach can be to adapt an existing formula. Ladas et al. used this approach in the most recent version of their formula, called the Ladas PLUS method. They utilized software (Python 3.7 with scikit-learn package) to improve a baseline method. Support vector regression, extreme gradient boosting, and an Artificial Neural Network were the supervised learning algorithms. Further, they performed a fivefold cross validation within the training set to prevent the overfitting of the model. This was obtained after randomly dividing the data into ten equal parts and subsequently utilizing nine of the ten to train the algorithm and testing it on the remaining tranche [17].

### 2.7. The PEARL-DGS Formula

In 2017, Debellemanière, Gatinel, and Saad proposed a new formula, developed using optical biometry and a machine learning model, called PEARL-DGS (DGS named after the formula developers) [23,40]. This is a thick lens method based on the forecast of the theoretical internal lens position (TILP), which is the theoretical distance from the posterior corneal surface to the anterior IOL surface, back-computed from postoperative data [17,23]. The TILP is independent of both the corneal thickness and the lens principal plane positions. This is forecasted utilizing different machine learning algorithms, e.g., neural networks, regular multiple regression, gradient boosted trees, and support vector regression. Additionally, Jupyter was utilized in the developing of the PEARL-DGS formula. This is an open-source application which allows the sharing of Python code in the form of a succession of cells, called Notebooks, facilitating research sharing and reproducibility. The corneal index was determined empirically during the formula development process, for the rest, the refractive indices of the Atchinson model eye were used [17]. The axial length was adjusted by the Cooke-modified AL formula (CMAL) [41]. The PEARL-DGS formula uses the following variables: AL, K, ACD, LT, WTW, and CCT [23]. Output linearization, adjustable IOL constants (similar to the third-generation formulas), and no re-training for new IOL models are typical of the PEARL-DGS method [9].

### 2.8. The Kane Formula

Jack X Kane, MD (Australia), presented his IOL power calculation formula in September 2017, using about 30,000 eyes. The method was developed utilizing several large data sets from chosen eye surgeons. It combines features of thin lens formulas, theoretical optics, and ‘big data’ techniques to make its predictions [12]. As a hybrid method, it is based on optics and incorporates both regression and AI components to further refine its prediction. Cloud-based computing was used to developed the formula. One focus of the formula was to improve the accuracy at the extremes of the various ocular dimensions where previous methods have yielded larger errors. The Kane formula requires the A-constant, AL, K, ACD, and patient biological sex as compulsory variables. Although adding the LT and CCT improves predictions, they are optional variables. Therefore, older biometers may be used to obtain biometric data. The formula has an A-constant very similar to the SRK/T A-constant. If the surgeon has an optimized A-constant, then that is recommended for use [42].

### 2.9. The Hoffer QST Formula

In 2021 Kenneth Hoffer (USA), Giacomo Savini (Italy), and Leonardo Taroni (Italy) developed the Hoffer QST formula, improving the third-generation vergence-based Hoffer Q by utilizing AI. Above all, they tried to increase its accuracy in long eyes. First, they aimed to improve the prediction of the ELP. For this purpose, they selected a totally independent training sample of 537 eyes. They entered into Linear Machine Learning model the following variables: K, AL, ACD measured from corneal epithelium to lens, corneal radius, and gender. The Python language with the Scikit-learn library was adopted and the best regressor of their cohort of data was the TheilSen model. As a second step, they developed a customized AL adjustment. Exploiting a reliable training data set of 375 eyes longer than 25.0 mm they optimized the individual AL using Excel. They then generated a new machine learning algorithm based on the above-mentioned inputs to find the ideal AL modification. This time the best regressor was the Huber model [25]. Their first results, presented during 2021 Annual Meeting of American Society of Cataract and Refractive Surgery, showed that the Hoffer QST formula improved the outcomes of Hoffer Q dramatically.

## 3. Results

**Figure 3:**
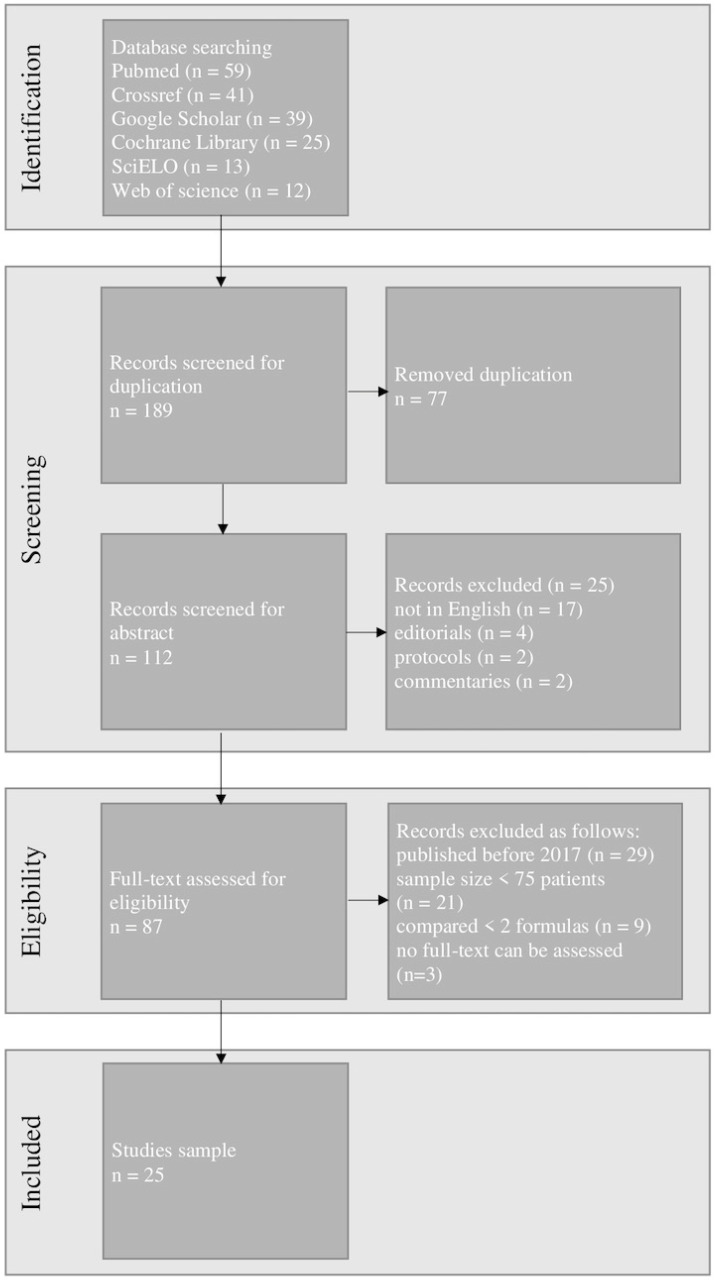
The Preferred Reporting Items for Systematic Reviews and Meta-Analysis (PRISMA) flow chart of our study.

The exclusion of duplicates resulted in 112 articles being retrieved and examined. After the initial search 25 articles were chosen for further study. Figure 3 shows the detailed PRISMA flow chart including the identification, screening, and selection of articles for this review.

The outcomes of the accuracy of IOL power calculation formulas based on AI in terms of MAE and percentage of eyes within ±0.5 D are summarized in Table 2 and Table 3.

In 2020, Darcy et al. published an analysis of 10,930 eyes implanted with four different IOL models: SA60AT (Alcon Laboratories, Inc.), Akreos Adapt AO (Bausch & Lomb, Inc. (Rochester, NY, USA)), Superflex Aspheric 920H, and C-Flex Aspheric 970C (Rayner Intraocular Lenses Limited (Worthing, UK)), and preoperative biometry was performed using partial coherence interferometry (IOLMaster; Carl Zeiss Meditec AG). They found that among the nine tested formulas Kane was the most accurate (MAE = 0.377; percentage of eyes within prediction error ≤±0.5 D = 72.0), ahead of Hill-RBF 2.0 (0.387 and 71.2, respectively). The third- and the fourth-generation formulas yielded worse outcomes [43].

Rocha-de-Lossada et al., in their 2020 study involving 171 eyes, considered 12 formulas, including 4 based on AI (Hill-RBF, Kane, PEARL-DGS, and Ladas) [2]. Considering the entire range of AL (*n* = 171). PEARL-DGS obtained the best outcomes, i.e., the lowest MAE (0.263) and the highest percentage of ±0.5 D (85.96%), while Ladas achieved the highest MAE (0.308) and the lowest percentage of ±0.5 D (81.29%). The results were very similar in medium eyes subgroup (22.5 mm < AL < 25.5 mm; *n* = 122). PEARL-DGS obtained the lowest MAE (0.263) and the highest percentage of ±0.5 D (86.89%), while Ladas obtained the highest MAE (0.313) and the lowest percentage of ±0.5 D (81.15%). However, in the short eyes subgroup (AL ≤ 22.5 mm; *n* = 42) Kane achieved the lowest MAE (0.237) and the highest percentage of ±0.5 D (90.7%), whereas Hill-RBF yielded the highest MAE (0.300) and Ladas the lowest percentage of ±0.5 D (83.72%). Due to the low sample size of long eyes, an analytical study of this subgroup has not been performed. It is worth noting that this study was conducted against Hoffer’s recommendations, because two eyes per patient were included [50].

The accuracy of 13 IOL power formulas (including 3 based on AI—Hill-RBF, Kane and PEARL-DGS) was considered by Hipólito-Fernandes et al. in their 2020 study comprising 828 eyes. Over the entire AL range, Kane achieved the best outcomes of the AI formulas. Moreover, of all the formulas considered Kane obtained the lowest MAE (0.324) and the highest percentage of ±0.5 D (79.3%). Contrarily, PEARL-DGS yielded the worst results—the highest MAE (0.344) as well as the lowest percentage of ±1.0 D (97.2%)—whereas Hill-RBF achieved the lowest percentage of eyes within ±0.5 D (76.7%). Moreover, Kane obtained the lowest MAE (0.348, 0.323 and 0.301, respectively) in all AL subgroups, e.g., short (AL ≤ 22.0 mm), medium (22.0 < AL < 26.0 mm), and long (AL ≥ 26.0 mm). On the other hand, PEARL-DGS achieved the highest MAE (0.368, 0.339, and 0.377, respectively) in all AL subgroups. However, the worse results of PEARL-DGS may be because it is optimized for values obtained with the IOLMaster700^®^ (Zeiss, Jena, Germany) whereas Hipólito-Fernandes et al. performed their preoperative optical biometry using optical low-coherence reflectometry (OLCR)—Lenstar LS-900^®^ (Haag-Streit AG, Köniz, Switzerland) [46].

Carmona-González et al., in their 2020 study, compared 11 IOL power calculation formulas, including 3 based on AI (Kane, Hill-RBF, and Ladas). Kane achieved the best results in the whole sample (*n* = 481 eyes), i.e., the lowest MAE (0.30) and the highest percentage of eyes within ±0.5 D (80.04%), whereas Hill-RBF was the worst, i.e., it had the highest MAE (0.33) and the lowest percentage of ±0.5 D (76.09%). On the other hand, Ladas had the highest risk of refractive surprise (3.12%), which was the lowest percentage of ±1.0 D (96.88%). It is worth pointing out that in medium-length eyes (22.0 mm < AL < 25.0 mm; *n* = 310) Ladas obtained the lowest MAE (0.29) as well as the highest percentage of ±0.5 D (81.29%), whereas Hill-RBF had the highest MAE (0.32) as well as the lowest percentage of all studied refractive subgroups (77.1% and 97.74%, respectively). In short (AL ≤ 22.0 mm; *n* = 57) and long eyeballs (AL ≥ 25.0 mm; *n* = 115) Kane achieved the lowest MAE (0.41 and 0.27, respectively) and the highest percentage of ± 0.5 D (66.67%; 86.84%, respectively). Hill-RBF obtained the lowest percentage of ±0.5 D (59.65) in short eyes, but the highest MAE (0.29) and the lowest percentage of ±0.5 D (81.58%) in long eyes. Ladas yielded the highest MAE (0.48) in short eyes. However, the biometric data utilized in these methods were all obtained with the IOLMaster 700, including keratometry. This means that one should be careful when extrapolating conclusions of the use of various biometric and keratometric measurement instruments [10].

Determining the exactness of 18 IOL power calculation formulas (including as many as 4 AI-based—Hill-RBF, Kane, Ladas, and PEARL-DGS) was the purpose of the 2021 study performed by Voytsekhivskyy et al. However, they included only short eyes with ALs ranging from 19.26 mm to 22.00 mm (*n* = 241). Kane achieved the lowest MAE (0.387) and the highest percentage of ±0.5 D (72.2%). On the other hand, Ladas obtained the highest MAE (0.468) and the lowest percentage of ±0.5 D (65.98%). The limitation of the study was the small number of eyes with an AL < 21.00 (34 eyes) and <20.00 (4 eyes) mm. However, these eyes are uncommon in the human population and thus a much larger time period is required to gather more patients. These numbers are inadequate for full-fledged statistical processing, which introduces some reductions in the credibility of the outcomes [12].

In 2023, Li et al. published their study on a database of 6893 eyes. The preoperative biometry records were obtained from Lenstar LS 900 optical biometers (Haag-Streit USA, EyeSuite software V.i9.1.0.0). Only a one-piece acrylic monofocal lens SN60WF (Alcon Laboratories, Inc.) was implanted. They realized that Nallasamy (MAE = 0.312; percentage of eyes within prediction error ≤±0.5 D = 80.2) outperformed seven other formulas (PEARL-DGS, Emmetropia Verifying Optical, Barrett Universal II, Haigis, SRK/T, Holladay 1, and Hoffer Q), though PEARL-DGS yielded promising results, i.e., 0.329 and 77.7, respectively [27].

Taroni et al. published, in 2023, their survey of 1259 eyes. They divided enrolled patients into two groups according to their race and IOLs as follows: White group (*n* = 696) with implanted Acrysof SN60AT (Alcon Laboratories, Inc.) and Asian group (*n* = 563) with Acrysof SN60WF (Alcon Laboratories, Inc.) Kane and HQST, of the AI-based formulas, obtained the best outcomes in the White group (MAE = 0.36; percentage of eyes within prediction error ≤±0.5 D = 76.29) while Hill-RBF 3.0 outperformed all other formulas in the Asian group (MAE = 0.26; percentage of eyes within prediction error ≤±0.5 D = 88.28). Overall, each formula obtained a better result in the Asian group than in the White group, however, the mean AL in the Asian group was longer than in the White group (23.94 mm and 23.50 mm, respectively) and the range was smaller (20.76–29.83 mm and 19.33–31.00 mm, respectively) [25].

## 4. Discussion

Various IOL power calculation formulas yield different outcomes. Many formulas have been developed over the years. Initially, these were simple mathematical equations. Subsequent generations of formulas have included more biometric parameters, which have resulted in greater accuracy [39]. Recently, formulas based on AI have been launched. The size of data sets is important here, and since they are still being updated the previously developed methods seem to be more accurate, e.g., Hill-RBF 3.0 is more precise than Hill-RBF 2.0 [51].

Several studies have recently compared the aforementioned methods as well as older formulas. Out of eight AI-based IOL power formulas only four have extensive representation in the literature: Hill-RBF [7,10,22,31], Kane [11,29,30,45], PEARL-DGS [9,12,21,46], and Ladas [2,7,10,44]. The other four formulas have been included in a few peer-reviewed articles. FullMonte is no longer operational [7], and Karmona [24], Hoffer QST [25], and Nallasamy were first published recently [27]. Among these four frequently published IOL power formulas, most peer-reviewed papers have considered two or three of them [7,22,30,32,43,47,48], and only two studies have included all four [2,12]. Table 4 shows precise data from selected articles.

To the authors’ best knowledge, this article is the first review that compares the latest studies of AI-based IOL power calculation formulas. A few other studies have been published on this topic (Savini et al., Xia et al., Guttierez et al.), but they covered fewer than 10 studies each, and no more than three articles since 2019 [52,53,54]. Our paper examines 23 studies performed after 2019 and the 25 total studies evaluate 32,648 operated eyes.

The review article by Savini et al. discusses new IOL power calculation methods based not only on AI but also formulas based on vergence and ray-tracing. They primarily show recent developments in implant power calculation; the comparison of formula accuracy is only a short section. They have included only a few studies where Kane, Hill-RBF, Ladas, and PEARL-DGS were considered [52].

The review by Xia et al. describes all currently used IOL power calculation formulas. They have concluded that AI formulas have great potential to further improve the accuracy of calculation, especially in short and long eyes [53]. Gutierrez et al. discuss the application of AI in cataract management. They tend to focus on the types of machine learning algorithms used in AI-based IOL power calculation formulas rather than on their accuracy [54]. A recently published review study by Stopyra et al. describes and compares most formulas on the market. They have observed that Barrett Universal II, among vergence, and Kane ahead of PEARL-DGS, among AI-based formulas, were most often shown as the most accurate [55].

This study has noteworthy limitations. First, we included the FullMonte IOL method, which is not currently used. However, it was the first IOL power calculation formula based on AI and as such inspired other authors to research in this area. Its importance in the development of AI-based IOL formulas was significant, although its accuracy was questionable. Second, we noted that the Karmona formula had no independent evaluation. However, we have tried to describe all the information on IOL formulas based on AI, even if it is biased. For the same reason, we included the Hoffer QST formula in the study, while realizing that it is described in only a few papers with a small sample size. We did not compare the results of the recently published Zhu-Lu formula because the only peer-reviewed article was concerned with only highly myopic eyes [56]. For this same reason we did not analyze the outcomes of the Zeiss AI formula. In this case, the only peer-reviewed paper was limited to short eyes [57]. Finally, although the Hill-RBF formula has three versions, the versions were not identified in our study for two reasons—firstly, it was impossible to identify a specific version of this formula in some articles, and secondly, the tables are much more readable if Hill-RBF is not divided into its different versions. No meta-analysis was performed, which was the most important limitation of this study. A meta-analysis is a valid, objective, and scientific method of analyzing and combining different results. However, the authors’ decision was fully thought-out. This decision was reached because there was too much heterogeneity in the included studies (different devices used to obtain biometric data, various implanted IOLs, a disparate range of biometric parameters such AL and ACD, varied equipment used to achieve postoperative refraction, etc.). Considering all these factors would significantly limit the statistical analysis; not considering them would result in bias. Finally, the protocol of this study was not prepared.

## 5. Conclusions

There is still no agreement among cataract surgeons regarding the choice of the ideal IOL power calculation formula. However, out of the AI-based formulas, Kane seems to be the most accurate. Many recent studies have shown that the Kane formula achieved the lowest MAE, and/or the highest percentage of eyes within ±0.5 D [10,12,22,28,29,30,31,32,43,44,45,48]. The authors observed the same in their original studies. Of the 12 examined formulas, Kane obtained the best outcomes in terms of MAE and percentage of ±0.5 D in the Stopyra study [58]. Similarly, Voytsekhivskyy et al. (Cooke, among others), in their study comprising 24 IOL power calculation formulas, showed that, among the methods based on AI, Kane yielded the highest percentage of ±0.5 D [59]. Hill-RBF, which is often regarded highly among the IOL formulas, achieved the lowest MAE only in Nemeth studies [6,11]. However, in many other studies it obtained the second-lowest outcome [7,9,10,12,22,28,29,32,43]. Only a few studies have shown the superiority of PEARL-DGS [2,9,21,26], however, recently, its online calculator was updated with a new version of the formula, so its results may be better in the future. Ladas obtained the best outcome only in a 2017 study and currently seems to be worse than other AI-based IOL formulas [7]. The superiority of Karmona was shown only by its author in a small sample of eyes [24]. It requires further examination. Initial results showed that Hoffer QST failed to yield more accurate predictions than other formulas (Shammas et al., 2022) [26], however, it outperformed the others in eyes which had undergone deep anterior lamellar keratoplasty [49].

Machine learning methods can be also incorporated into a variety of existing IOL power formulas to refine their accuracy [60,61]. Overall, IOL power calculation formulas based on AI are promising and have the potential to positively affect the accuracy of postoperative refractions after cataract surgery. It can be expected that newer and more exact AI-based methods will be developed. And certainly, the most frequently used formulas today will be upgraded into new versions in the future.

## Figures and Tables

**Table 1:** Variables utilized in formulas based on artificial intelligence.

Formula		Logical Approach Classification	Variables Utilized								
			AL	K	KmA	KmP	ACD	LT	CCT	WTW	G
FullMonte		Hybrid	C	C			C	C			
Ladas		Hybrid	C	C			C				
Hill-RBF	1.0	AI	C	C			C				
	2.0		C	C			C				
	3.0		C	C			C	O	O	O	O
PEARL-DGS		Hybrid	C	C			C	C	C	C	
Kane		Hybrid	C	C			C	O	O		C
Karmona		AI	C		C	O	C	O		C	C
Hoffer QST		Hybrid	C	C			C				C
Nallasamy		AI	C	C			C	C	O	C	

AL = axial length; K = keratometry; KmA = keratometry-mean anterior; KmP = keratometry-mean posterior; ACD = anterior chamber depth; LT = lens thickness; CCT = central corneal thickness; WTW = white to white; G = gender; RBF = radial basis function; PEARL = Prediction Enhanced by ARtificial Intelligence and output Linearization; DGS = Debellemanière, Gatinel, Saad; Hoffer QST = Hoffer Q, Savini, Taroni; C = compulsory; O = optional, AI = artificial intelligence.

**Table 2:** Outcomes of absolute error in several of the latest studies.

Study	MAE							
	Hill-RBF	Karmona	Nallasamy	Kane	Ladas	Hoffer QST	PEARL-DGS	FullMonte
Kane et al., 2017; (3122 eyes) [7]	0.407				0.402			0.428
Connell and Kane, 2019; (864 eyes) [22]	0.346			0.329				
Darcy et al., 2020; (10,930 eyes) [43]	0.387			0.377				
Carmona-González et al., 2020; (481 eyes) [10]	0.33			0.30	0.32			
Hipólito-Fernandes et al., 2020; (695 eyes) [21]	0.337			0.315			0.312	
Savini et al., 2020; (200 eyes) [31]	0.287			0.265			0.286	
Rocha-de-Lossada et al., 2020; (171 eyes) [2]	0.283			0.276	0.308		0.263	
Cheng et al., 2020; (370 eyes) [29]	0.46			0.34				
Kane and Melles, 2020; (182 eyes) [28]	0.709			0.533				
Nemeth and Modis, 2020; (186 eyes) [6]	0.36							
Tan et al., 2020; (111 eyes) [44]				0.72	0.74			
Hou et al., 2021; (129 eyes) [32]	0.44			0.41	0.51			
Carmona-González et al., 2021; (260 eyes) [24]	0.3	0.24						
Ang et al., 2021; (183 eyes) [30]				0.34	0.39			
Nemeth et al., 2021; (114 eyes) [11]	0.36			0.42			0.41	
Zhang et al., 2021; (211 eyes) [45]				0.55	0.57			
Hipólito-Fernandes et al., 2021; (828 eyes) [46]	0.342			0.324			0.344	
Voytsekhivskyy et al., 2021; (241 eyes) [12]	0.413			0.387	0.468		0.421	
Hipólito-Fernandes et al., 2021; (220 eyes) [47]				0.364			0.377	
Debellemanière et al., 2021; (4242 eyes) [9]	0.303						0.286	
Chang et al., 2022; (79 eyes) [48]				0.40			0.46	
Shammas et al., 2022; (595 eyes) [26]	0.31			0.30		0.31	0.29	
Pellegrini et al., 2022; (82 eyes) [49]				1.10		1.05		
Taroni et al., 2023 (1259 eyes) [25]	0.38			0.36		0.36		
Li et al., 2023 (6893 eyes) [27]			0.31				0.33	

**Table 3:** The percentage of eyes within ±0.5 D.

Study	% ±0.5 D							
	Hill-RBF	Karmona	Nallasamy	Kane	Ladas	Hoffer QST	PEARL-DGS	FullMonte
Kane et al., 2017; (3122 eyes) [7]	69.6				69.8			66.6
Connell and Kane, 2019; (864 eyes) [22]	75.3			77.9				
Darcy et al., 2020; (10930 eyes) [43]	71.2			72.0				
Carmona-González et al., 2020; (481 eyes) [10]	76.1			80.0	79.8			
Hipólito-Fernandes et al., 2020; (695 eyes) [21]	77.9			81.6			79.9	
Savini et al., 2020; (200 eyes) [31]	85.0			86.5			84.5	
Rocha-de-Lossada et al., 2020; (171 eyes) [2]	85.4			84.8	81.3		86.0	
Cheng et al., 2020; (370 eyes) [29]	69.5			75.0				
Kane and Melles, 2020; (182 eyes) [28]	44.0			58.5				
Nemeth and Modis, 2020; (186 eyes) [6]	83.6							
Tan et al., 2020; (111 eyes) [44]				59.8	49.5			
Hou et al., 2021; (129 eyes) [32]	69.0			71.3	60.5			
Carmona-González et al., 2021; (260 eyes) [24]	80.8	90.4						
Ang et al., 2021; (183 eyes) [30]				80.9	75.4			
Nemeth et al., 2021; (114 eyes) [11]	84.2			80.7			79.8	
Zhang et al., 2021; (211 eyes) [45]				65.9	64.5			
Hipólito-Fernandes et al., 2021; (828 eyes) [46]	76.7			79.3			76.9	
Voytsekhivskyy et al., 2021; (241 eyes) [12]	67.7			72.2	66.0		68.5	
Hipólito-Fernandes et al., 2021; (220 eyes) [47]				71.6			74.1	
Debellemanière et al., 2021; (4242 eyes) [9]							87.4	
Chang et al., 2022; (79 eyes) [48]				70.9			65.8	
Shammas et al., 2022; (595 eyes) [26]	81.5			80.3		80.7	81.7	
Pellegrini et al., 2022; (82 eyes) [49]				32.5		37.0		
Taroni et al., 2023 (1259 eyes) [25]	74.7			76.3		76.3		
Li et al., 2023 (6893 eyes) [27]			80.2				77.7	

**Table 4:** Outcomes of mean absolute error, % of ±0.5 D, and % of ±1.0 D.

Study	Limitations	Parameter	Formula							
			Hill	Karm	Nall	Kane	Ladas	HQST	DGS	FullM
Rocha-de-Lossada et al., 2021; 171 eyes [2]	Small sample size, two eyes per patient included (against Hoffer recommendation) (Hoffer et al., 2015) [50] Phaco performed by one surgeon	MAE	0.28			0.28	0.31		0.26	
		±0.5 D	85.4			84.8	81.3		86.0	
		±1.0 D	98.8			100	98.3		100	
Shammas et al., 2022; 595 eyes [26]	Small sample size of short and long eyes Lack of extremely short and long eyes Biometry with the Argos (Alcon, Fort Worth, TX, USA) biometer (sum-of-segment methodology for AL)	MAE	0.31			0.30		0.31	0.29	
		±0.5 D	81.5			80.3		80.7	81.7	
		±1.0 D	99.5			99.7		99.8	99.8	
Hipólito-Fernandes et al., 2020; 828 eyes [46]	The same model of implanted IOL Preoperative optical biometry with optical low-coherence reflectometry (OLCR)—Lenstar LS- 900^®^ (Haag-Streit AG, Köniz, Switzerland)	MAE	0.34			0.32			0.34	
		±0.5 D	76.7			79.3			76.9	
		±1.0 D	97.6			97.7			97.2	
Carmona-González et al., 2021; 481 eyes [10]	Small sample size of short and long eyes	MAE	0.33			0.30	0.32			
		±0.5 D	76.1			80.0	79.8			
		±1.0 D	97.5			98.1	96.9			
Kane et al., 2017; 3122 Eyes [7]	Single IOL model (Hill-RBF and Barrett Universal II have been developed or reformulated based on data using this particular IOL type) Lack of full optimization of the lens constant for each formula	MAE	0.41				0.40			0.42
		±0.5 D	69.6				69.8			66.6
		±1.0 D	94.3				94.3			93.0
Voytsek hivskyy et al., 2021; 241 eyes [12]	Only short eyes (lack of medium and long eyes) Small sample size of very short eyes	MAE	0.41			0.39	0.47		0.42	
		±0.5 D	67.7			72.2	66.0		68.5	
		±1.0 D	93.8			95.4	88.4		93.8	
Savini et al., 2020; 200 eyes [31]	Lack of LT data does not show the full capabilities of formulas that can utilize this parameter, such as Kane, Pearl-DGS, and Hill-RBF Small sample size Phaco performed by one surgeon	MAE	0.29			0.27			0.29	
		±0.5 D	85.0			86.5			84.5	
		±1.0 D	99.5			99.0			98.5	
Darcy et al., 2020; 10,930 [43] eyes	Absence of LT, CCT, and WTW measurements	MAE	0.39			0.38				
		±0.5 D	71.2			72.0				
		±1.0 D	94.9			95.2				
Carmona- González et al., 2021; 260 eyes [24]	Small sample size	MAE	0.30	0.24						
		±0.5 D	80.8	90.4						
		±1.0 D	100	100						
Zhang et al., 2021; 211 eyes [45]	Only vitrectomized eyes (relatively worse visual acuity)	MAE				0.55	0.57			
		±0.5 D				65.9	64.5			
		±1.0 D				85.8	85.3			
Hipólito- Fernandes et al., 2021; 220 eyes [47]	Retrospective design of the study	MAE				0.36			0.38	
		±0.5 D				71.6			74.1	
		±1.0 D				95.7			95.7	
Taroni et al., 2023; 1259 eyes [25]	Lack of LT data does not show the full capabilities of formulas that can utilize this parameter, such as Kane and Hill-RBF Retrospective design of the study	MAE	0.38			0.36		0.36		
		±0.5 D	74.7			76.3		76.3		
		±1.0 D	95.0			96.0		96.7		
Li et al., 2023; 6893 eyes [27]	No validation on a data set from a different institution Single IOL model, i.e., SN60WF (Alcon, Fort Worth, TX, USA) Small range of AL (no extremely long or extremely short eyes)	MAE			0.31				0.33	
		±0.5 D			80.2				77.7	
		±1.0 D			97.6				97.4	

## Data Availability

No new data were created or analyzed in this study. Data sharing is not applicable to this article.
